# Proximal migration of a biliary stent: what to do after failure of conventional and cholangioscopic retrieval techniques

**DOI:** 10.1016/j.vgie.2020.12.007

**Published:** 2021-02-06

**Authors:** Zain A. Sobani, Nida S. Bham, Tarun Rustagi

**Affiliations:** 1 Division of Gastroenterology and Hepatology, University of New Mexico, Albuquerque, New Mexico; 2 Department of Surgery, University of New Mexico, Albuquerque, New Mexico

Removal of biliary stents that have migrated proximal to a stricture is especially challenging. Here we demonstrate a novel method to retrieve such stents after failure of conventional and cholangioscopic techniques.

A 57-year-old man initially presented with obstructive jaundice. He underwent an ERCP which demonstrated a hilar stricture and bilateral plastic stents were placed. Follow-up ERCP for planned stent change and single-operator cholangioscopy-guided biopsy revealed proximal migration of the left duct stent (Fig. 1A and B). Attempts to retrieve the stent with an extraction balloon (9-12 mm, Extractor Pro XL, Boston Scientific, Marlborough, Mass, USA) and basket (2 cm trapezoid basket, Trapezoid RX, Boston Scientific) were unsuccessful. Attempts to advance the guidewire into the stent under fluoroscopy were also unsuccessful.

**Figure 1:**
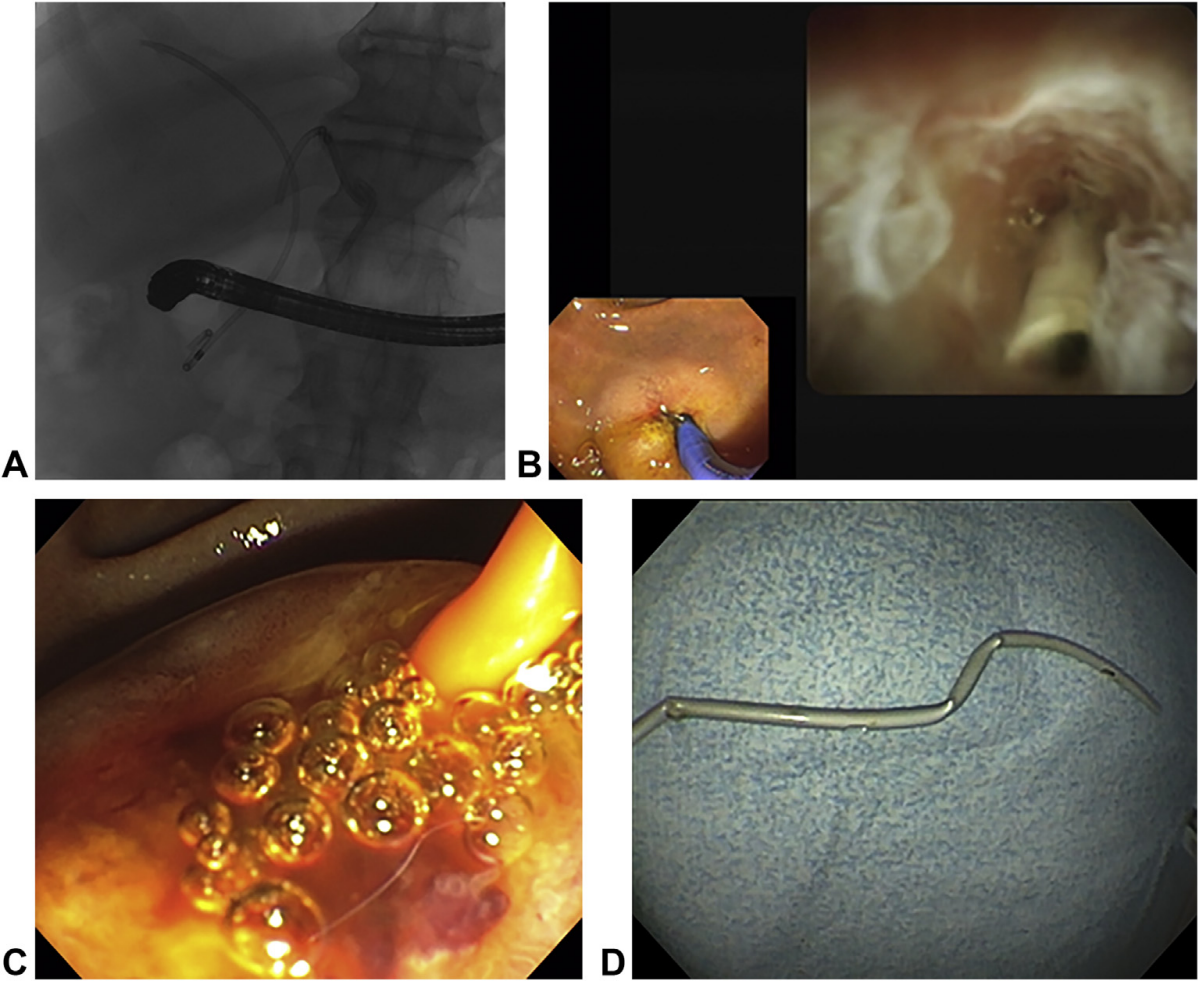
**A,** Fluoroscopic view showing the right duct stent in place whereas the left duct stent was noted to have migrated proximally within the bile duct. **B,** Cholangioscopy showing the distal end of the stent partially embedded within the stricture. **C,** The low-profile angioplasty balloon engaged within the stent. **D,** The successfully removed stent.

Single-operator cholangioscopy was performed using a SpyScope DS II cholangioscope (Boston Scientific) (Video 1, available online at www.giejournal.org). The distal end of the stent was noted to be partially embedded within the stricture. Attempts to remove the stent using SpyBite biopsy forceps, SpyGlass retrieval basket, and SpyGlass retrieval snare (Boston Scientific) under cholangioscopic guidance were unsuccessful. Given that the distal end of the stent was embedded within the stricture, it created a shelf like effect leading to failure of conventional and cholangioscopic techniques to adequately grasp the stent.

Next, a 0.025-inch straight-tip Visiglide 2 guidewire (Olympus Medical, Center Valley, Pa, USA) was advanced through the cholangioscope into the stent. Attempts to pass a 4-mm biliary dilating balloon catheter (Fusion Titan, Cook Medical, Bloomington, Ind, USA) into the stent were unsuccessful and resulted in further proximal migration of the stent. A 3–4–5F ultra-tapered tip catheter (Contour ERCP Cannula, Boston Scientific) was advanced into the stent, and the guidewire was exchanged for a 0.018-inch angled gold-tipped Terumo guidewire (Terumo Corporation, Shibuya City, Tokyo, Japan). A low-profile percutaneous transluminal angioplasty balloon catheter (balloon size: 3 mm diameter × 40 mm length; catheter length: 170 cm; Advance 18 LP, Cook Medical) was successfully advanced over the 0.018-inch guidewire into the stent given its low-profile (4F) catheter. This percutaneous transluminal angioplasty balloon designed for obstructive lesions in peripheral arteries accepts a 0.018-inch guidewire and is typically used in the endoscopy unit to dilate very high-grade, difficult-to-traverse pancreaticobiliary strictures. The balloon was inflated within the stent, and the stent was successfully pulled down and retrieved (Fig. 1C and D).

This technique of using a low-profile angioplasty balloon over a 0.018-inch guidewire inserted into the stent under cholangioscopic guidance is an effective method of retrieving a proximally migrated stent after failure of conventional and cholangioscopic techniques.

## Disclosure

*Dr Rustagi is a consultant for Boston Scientific. All other authors disclosed no financial relationships.*

## Supplementary data

Video 1 Novel method to retrieve a proximally migrated biliary stent after failure of conventional and cholangioscopic techniques.

